# Soil bacterial community composition is more stable in kiwifruit orchards relative to phyllosphere communities over time

**DOI:** 10.1186/s40793-023-00526-5

**Published:** 2023-08-24

**Authors:** Ziva Louisson, Louis Ranjard, Hannah L. Buckley, Bradley S. Case, Gavin Lear

**Affiliations:** 1 School of Biological Sciences, University of Auckland, 3a Symonds Street, Auckland, 1010 New Zealand; 2 PlantTech Research Institute, 29 Grey St, Tauranga, 3011 New Zealand; 3 School of Science, Auckland University of Technology, 34 St Paul Street, Auckland, 1010 New Zealand

**Keywords:** Temporal change, Microbiome, 16S rRNA gene, Community assembly processes

## Abstract

**Background:**

Soil and phyllosphere (leaves and fruit) microbes play critical roles in the productivity and health of crops. However, microbial community dynamics are currently understudied in orchards, with a limited number incorporating temporal monitoring. We used 16S rRNA gene amplicon sequencing to investigate bacterial community temporal dynamics and community assembly processes on the leaves and fruit, and in the soil of 12 kiwifruit orchards across a cropping season in New Zealand.

**Results:**

Community composition significantly differed (*P* < 0.001) among the three sample types. However, the communities in the phyllosphere substrates more closely resembled each other, relative to the communities in the soil. There was more temporal stability in the soil bacterial community composition, relative to the communities residing on the leaves and fruit, and low similarity between the belowground and aboveground communities. Bacteria in the soil were more influenced by deterministic processes, while stochastic processes were more important for community assembly in the phyllosphere.

**Conclusions:**

The higher temporal variability and the stochastic nature of the community assembly processes observed in the phyllosphere communities highlights why predicting the responsiveness of phyllosphere communities to environmental change, or the likelihood of pathogen invasion, can be challenging. The relative temporal stability and the influence of deterministic selection on soil microbial communities suggests a greater potential for their prediction and reliable manipulation.

**Supplementary Information:**

The online version contains supplementary material available at 10.1186/s40793-023-00526-5.

## Introduction

Rhizosphere and phyllosphere microbes form tight associations with host plants, both mutualistic and antagonistic [7], with plant-associated microbes performing beneficial roles, including plant growth promotion [1, 9], stress tolerance [4], disease suppression [21, 82] and nutrient cycling [2, 52]. Plant microbiome composition and dynamics are shaped by host-microbe interactions, including the plants defense systems [28] and phytochemical production [15, 73], such as phytohormones [20]. The rhizosphere and phyllosphere are spatially distinct plant habitats. The rhizosphere, which surrounds the plant root, is a highly complex and nutrient-rich environment that is influenced by the roots and harbours an abundance of diverse microorganisms [61]. In contrast, the phyllosphere, which describes the aerial components of the plant microbiome, is considered a more nutrient-limited environment, exposed to more variable conditions than the soil, such as fluctuations in temperature, moisture and UV radiation [73]. Relative to soil environments, the phyllosphere has low microbial abundance [23]. However, phyllosphere communities still play an important role in plant health and it is a potential entry point for bacterial pathogens [19].

A focus in microbial ecology is identifying the underlying drivers that shape microbial community structure. The physiochemical properties of soil environments are highly influenced by land use and land management practices, which in turn strongly impact the soil bacterial community composition [29–31]. While less research has been directed towards the phyllosphere, primary sources of phyllosphere microbes have been identified as soils, plant seeds, the atmosphere and insect vectors [6, 24, 49]. Some studies have identified that soil is the dominant source of phyllosphere microbes, perhaps acting as a bacterial reservoir [24, 85], while others suggest that the atmospheric microbes are important for phyllosphere colonization [49, 56]. As the plant microbiome is increasingly linked to ecosystem functioning, there are growing efforts to understand the dynamics of microbial communities in different agricultural systems, and particularly their consequences for plant health [24, 67, 85].

Understanding the mechanisms shaping microbial community assembly through resolving the relative influence of deterministic and stochastic processes on community structure is an important theme in microbial ecology [25, 53, 88]. Knowledge of these mechanisms is also of importance for managing plant health, since deterministic influences are intrinsically easier to predict than stochastic drivers of community change. Deterministic theories specify that species patterns are shaped mainly by selection imposed by the abiotic environment and biotic interactions [70]. Under similar and consistent environmental conditions, selection leads to a higher-than-expected similarity in community composition between local communities (homogeneous selection), while under variable environmental conditions, selection leads to lower-than-expected similarity in community composition between local communities (heterogeneous selection) [70, 76]. In contrast, stochastic processes involve less predictable factors such as dispersal, the movement of individuals from one location to another leading to chance colonization [17], and ecological drift and random extinction. When two local communities reside in environments with distinct abiotic conditions, but have high rates of dispersal between them, this ‘homogenising dispersal’ can lead to communities having more similar composition than would otherwise be expected. In contrast, if two environments have similar abiotic conditions, but low dispersal rates, this ‘dispersal limitation’ can assist to maintain community compositions that are more distinct than might otherwise be expected. However, dispersal limitation alone does not solely drive community composition turnover, but rather heightens the influence of stochastic changes (drift) in population size and spatial turnover [70, 76]. In addition, random births and deaths, rather than deterministic factors, can contribute to greater differences among communities via ‘ecological drift’ [53].

Stegen et al. [68] developed an analytical framework to infer the relative contributions of selection, dispersal and drift in structuring microbial communities. The framework is a two-step null model workflow based on phylogenetic and taxonomic turnover. Application of this framework to determine the balance between these ecological processes has been explored across various environments, including marine waters [78], lakes [3, 47], soil [35, 71] and crop systems [45, 87]. Community assembly processes have been explored in phyllosphere communities; however, research has focused mainly on examining primary colonisation sources [24] or successional dynamics in community structure [49]. Research quantifying the relative influence of specific assembly processes (i.e., selection, dispersal and drift) on bacterial community structure among different plant components and how they vary through time remains largely unexplored. Soil pH and plant exudates are identified as important factors influencing the balance of deterministic and stochastic assembly processes in soil and plant-associated microbial communities [71, 72, 86], however, less is known about the influence of underlying environmental factors on community assembly in managed agricultural systems. Understanding community assembly processes and the factors regulating such processes in agricultural systems can provide insights into the strength of the relationship between abiotic conditions and ecosystem processes, and the degrees to which communities can be influenced by targeted land management, such as inoculum transplants [8].

This study examines the temporal dynamics and community assembly processes of phyllosphere and soil bacterial communities in kiwifruit orchards, which are known to be vulnerable to invasion by pathogens such as *Pseudomonas syringae* pv. *actinidiae* (Psa). Disentangling the interactions between kiwifruit soil and phyllosphere microbial communities and understanding how the dynamics of these communities change through time can improve our understanding of plant–microbe interactions and form the basis for potential avenues for integrating crop-associated microbes into orchard management and protection. To our knowledge, this is among the first studies to examine temporal variation in the kiwifruit microbiome over a cropping season. We used 16S rRNA gene amplicon sequencing to characterise differences in the structure of leaf, fruit and soil bacterial communities and examine how they change over time. We analysed bacterial communities from 12 kiwifruit orchards at six times, across the cropping season from December to June. We investigate the following questions: (1) Does the composition of bacterial communities vary among substrates? (2) Do the microbial communities in the different components of the plant exhibit different levels of stability? (3) How similar are the aboveground and belowground communities? (4) What are the dominant mechanisms driving bacterial community composition in each substrate? Investigating these questions will help us better understand the interconnectedness of crop microbiomes and further our understanding of the fundamental ecological processes driving community structure. A thorough understanding of crop microbiome diversity and dynamics could aid us in integrating beneficial microbes into promoting agricultural production.

## Materials and methods

### Site description and sample collection

We collected soil, leaf and fruit samples from 12 kiwifruit orchards in the Bay of Plenty region of New Zealand, including orchards of green (Haywards) and gold (Hort16A, Gold3) cultivars, the latter of which has greater resistance to the Psa pathogen [75]. The Hayward orchards have an age range of 20 to 45 years, while the Gold orchards are aged between 15 to 30 years. The orchards are representative sites from New Zealand’s dominant kiwifruit growing zone, with an average total precipitation of 623.2 mm ± 20.7 and mean annual temperatures of 13.7 °C ± 0.15 °C [83]. Sampling was conducted six times throughout an entire season, with samples collected in December 2020, January, March, April, May and June 2021. However, we did not collect fruit samples for the January sampling due to insufficient resources at the time. Five kiwifruit vines were selected per site, with the same vines sampled at each time for soil (n = 360), leaves (n = 360) and fruit (n = 300). Six soil cores (0–10 cm depth, 2.5 cm diameter) were taken 1 m from the base of each vine in a radial pattern. At each sampling time, the soil cores were manually homogenised and pooled to form a composite sample per vine. To sample the phyllosphere, five different canes from each vine were selected, and ten fully developed leaves (two per cane) and five fruit (one per cane) were cut with sterile snips. The leaves and the fruit from each vine were composited to form one leaf and one fruit sample per vine, per sampling time. All samples were placed into sterile bags and kept on ice during sample collection, until they were transferred to − 20 °C storage, awaiting DNA extraction. Soil samples, composited per orchard for the first sampling occasion (December 2020), were used to determine soil chemical properties for each site (Additional file 1: Table S1).

## DNA extraction, PCR and sequencing

To extract phyllosphere, or surface-associated DNA, five leaves were chosen from the ten leaf composite samples (per vine) and washed for 30 s in 50 mL of sterile isotonic saline (0.90% w/v of NaCL, 9.0 g per L) containing 0.01% Tween 80. All five fruits collected per vine were similarly washed. For each composite sample, 25 mL of the washing liquid was transferred to 15 mL falcon tubes and centrifuged at 4000 ×*g* for 20 min. The supernatant was discarded, and the falcon tubes were stored at − 20 °C. Before DNA extraction, the material from the washed leaves and fruits were thawed and resuspended in 200 µl of sterile water; the soil cores were thawed and manually homogenized. We extracted genomic DNA from 250 mg of each soil sample and the 200 µl of each leaf and fruit wash liquid using DNeasy PowerSoil HTP kits (Qiagen, Valencia, CA, USA) according to the manufacturer’s instructions, with the following modifications; (i) a Qiagen TissueLyer II instrument (Retch) was used for mechanical lysis for 4 min at a frequency of 30 Hz; (ii) after adding the elution buffer, the plates were incubated at room temperature for 5 min. The extracted DNA (360 soil, 360 leaf, 300 fruit = 1020 samples) was placed in − 20 °C storage. We amplified the V3/V4 region of 16S ribosomal RNA genes through PCR using the primers 341F (5′-TCGTCGGCAGCGTCAGATGTGTATAAGAGACAGCCTACGGGNGGCWGCAG-3′) and 785R (5′-GTCTCGTGGGCTCGGAGATGTGTATAAGAGACAGGACTACHVGGGTATCTAATCC-3′), modified with an overhang region complementary to the Illumina DNA sequencing adapters (underlined), which were optimised for use on Illumina MiSeq platforms [42]. Amplification reactions were carried out in a final volume of 25 µl containing 2.5 µl of 10X MTP Taq Buffer, 0.5 µl of deoxynucleotide triphosphate mixture (dNTP; 10 mM), 0.75 µl of each primer (10 µM) and 0.25 µl of MTP™ Taq DNA polymerase (Sigma-Aldrich, France). DNA template was amplified from each sample using the following conditions: (i) initial denaturation at 95°C for 3 min; (ii) 25 cycles of denaturation at 95°C for 30 s, annealing at 55°C for 30 s, and extension at 72°C for 30 s; and then (iii) a final extension at 72°C for 5 min. We purified the PCR products using DNA clean-up kits (Zymo Research), according to the manufacturer’s instructions. DNA concentration was quantified using a Qubit double-stranded DNA HS assay kit (Thermo Fisher Scientific) and samples were normalised to 4 ng/µl. The purified PCR products were submitted to New Zealand Genomics Ltd. (Auckland, New Zealand), which were then barcoded (Nextera XT dual indices), pooled and sequenced on an Illumina MiSeq instrument, producing 2 × 300 bp paired-end reads. A ZymoBIOMICS Microbial Community Standard (mock extraction standard) and a ZymoBIOMICS Community DNA Standard (mock community standard) were used as controls to examine DNA isolation and sequencing run bias, respectively (Zymo Research). We extracted DNA from the mock extraction standard in the same manner as our samples. We amplified the isolated DNA from the mock extraction and community standard. We included the amplicons from the mock extraction standard on two sequencing runs and those from the mock community standards on all six sequencing runs.

## Bioinformatics and statistical analysis

Demultiplexed sequence data were processed in R v 4.2.2 [64], following the DADA2 pipeline [13]. We identified exact amplicon sequence variants (ASVs) using the DADA2 algorithm, resolving sequences at a single-nucleotide level. Compared to traditional clustering approaches, DADA2 enables increased accuracy in identifying real biological variants and greater reproducibility [12]. Using the DADA2 package, we trimmed and filtered the reads to remove low-quality reads and primers, merged the paired-end reads and removed chimeras. For taxonomic assignment, we used the RDP’s naïve Bayesian classifier method [79] and the SILVA rRNA gene database, version 138.1 [63]. We omitted ASVs not categorised as bacterial, or those classified as chloroplasts or mitochondria. We rarefied the reads to 7,000 sequence reads per sample (Additional file 1: Fig S1) for alpha diversity analyses, using the *rarefy_even_depth* function in the ‘phyloseq’ package [51]. After rarefaction, we retained 1001 out of 1020 samples. For beta-diversity analyses, all samples with < 1000 reads were removed, as biological patterns can be obscured by low library sizes, as recommended by Weiss et al., [81]. We retained 1011 out of 1020 samples and Cumulative Sum Scaling (CSS) normalisation was executed on the non-rarefied data as recommended [58]. Differences were tested for statistical significance using a permutational multivariate analysis of variance (PERMANOVA), implemented with the *adonis* function in the ‘vegan’ package [54]. For the CSS normalised data, the replicate data were averaged for each site and time to generate one representative bacterial community per substrate, site and sampling time, which was used for all downstream analyses, unless stated otherwise. To construct a phylogenetic tree, the sequences were aligned using MAFFT (v 7.505) [36] with default settings, trimmed using trimA1 (v 1.2 rev 59) [14] with parameters set to -gt 0.3 and -st 0.001. The tree was constructed using FastTree (v 2.1.10) [62] with -gtr and -nt options selected. Climatic data were extracted for each site using GIS software from NIWA interpolations of climate station data [83]. We identified soil chemical and climatic variables which strongly correlated with each other (Pearson’s correlation < − 0.6 or > 0.6), and selected one of the variables as representative for downstream analyses. We retained three climatic variables and six soil chemical variables and discarded four and nine variables, respectively (Additional file 1: Table S2).

## Sequencing summary

In the mock communities, taxonomy was assigned to genus levels and all eight of the bacterial genera defined in the mock communities were correctly identified in our mock samples (*Lactobacillus* was assigned as *Limosilactobacillus*); no other genera were identified (Additional file 1: Fig S2). The mock extraction indicated some bias against Gram-positive bacteria, with lower relative abundances of *Enterococcus* and *Listeria*. This extraction bias has been previously observed and is attributed to their thick cell wall [26]. The mock community standard displayed a comparable community structure across five sequencing runs. The mock sample from sequencing run three was omitted as there were no sequencing reads for this sample. Two negative samples, consisting of a pooled negative control from each PCR master mix and a control from the extraction kit, were also included in a sequencing run. We used the *isContaminant* function from the R package ‘Decontam’ [18] to identify and remove contaminant sequences. Eight taxa were identified as contaminants in the control sample and these taxa were removed for downstream analyses.

## Quantitative analyses

To analyse the alpha diversity of communities from the different substrates (leaf, fruit and soil) and sampling times, we computed Shannon diversity index values using the *estimate_richness* function in ‘phyloseq’ [51]. For each substrate, we assessed the effects of orchard and time on the Shannon diversity index using analysis of variance (ANOVAs). For each substrate, we tested for statistically significant differences in Shannon diversity among sampling times, using a Dunn’s test. We compared the bacterial communities among the different substrates and times by computing Bray–Curtis dissimilarity matrices using the ‘vegan’ package [54] in ‘R’ [64]. To investigate the relationship between the soil and phyllosphere communities, we quantified the proportion of ASVs uniquely found in each substrate and the proportion of ASVs shared with the other substrates for the leaf, fruit and soil communities. We plotted the proportion of unique and shared taxa for each substrate and sampling date. We excluded data from January as we do not have fruit community data for this date. We visualised the differences in community composition between substrates and times using non-metric multidimensional scaling (NMDS). Differences were tested for statistical significance using a PERMANOVA, implemented with the *adonis* function in the ‘vegan’ package. Centroids of data representing each site, sampling date, substrate and kiwifruit variety (Gold or Hayward) were calculated and an NMDS plot was generated to visualise the Bray–Curtis dissimilarity distance between the centroids. To investigate the temporal trajectory of change for each substrate individually, the centroid of data representing each sampling date was calculated separately for the leaf, fruit and soil data and visualised on an NMDS plot. We performed a Mantel test, using the *mantel* function [54], to investigate the relationship between bacterial community composition (Bray–Curtis dissimilarity) and geographical distance (Euclidean distance between orchard coordinates).

## Quantification of community assembly processes

We used the non-averaged replicate data to infer ecological processes, running analyses separately for each substrate and time (n = 1020; n = 60 per substrate and time). We applied a two-step null model approach, described by [68, 69], to infer the degree to which the leaf, fruit and soil communities are influenced by homogeneous selection, heterogeneous selection, dispersal limitation, homogenising dispersal and drift, and how this changes through time. A requirement of the framework is that closely related taxa share habitat preferences, as determined by testing for phylogenetic relatedness. The weighted average score of the soil chemical and climatic variables was calculated for each ASV, and a Euclidean distance matrix was generated to represent differences in niche optima between ASVs. The *cophenetic.phylo* function in the ‘ape’ package [57] was used to compute a phylogenetic distance matrix based on the branch lengths of the phylogenetic tree. A Mantel correlogram was generated to relate niche differences between ASVs to their phylogenetic distances, using the *mantel.correlog* function from the ‘vegan’ package in ‘R’ [54]. The significance of these correlations was tested using 999 permutations of the data, with Holm correction to counteract the problem of multiple comparisons [32]. Phylogenetic clustering was observed across very short phylogenetic distance, indicating ecological niche distance increases with phylogenetic distance (Additional file 1: Fig S3). Phylogenetic overdispersion was observed at slightly more distant phylogenetic distances. These patterns became non-significant over larger phylogenetic distances (Additional file 1: Fig S3).

**Fig. 1:**
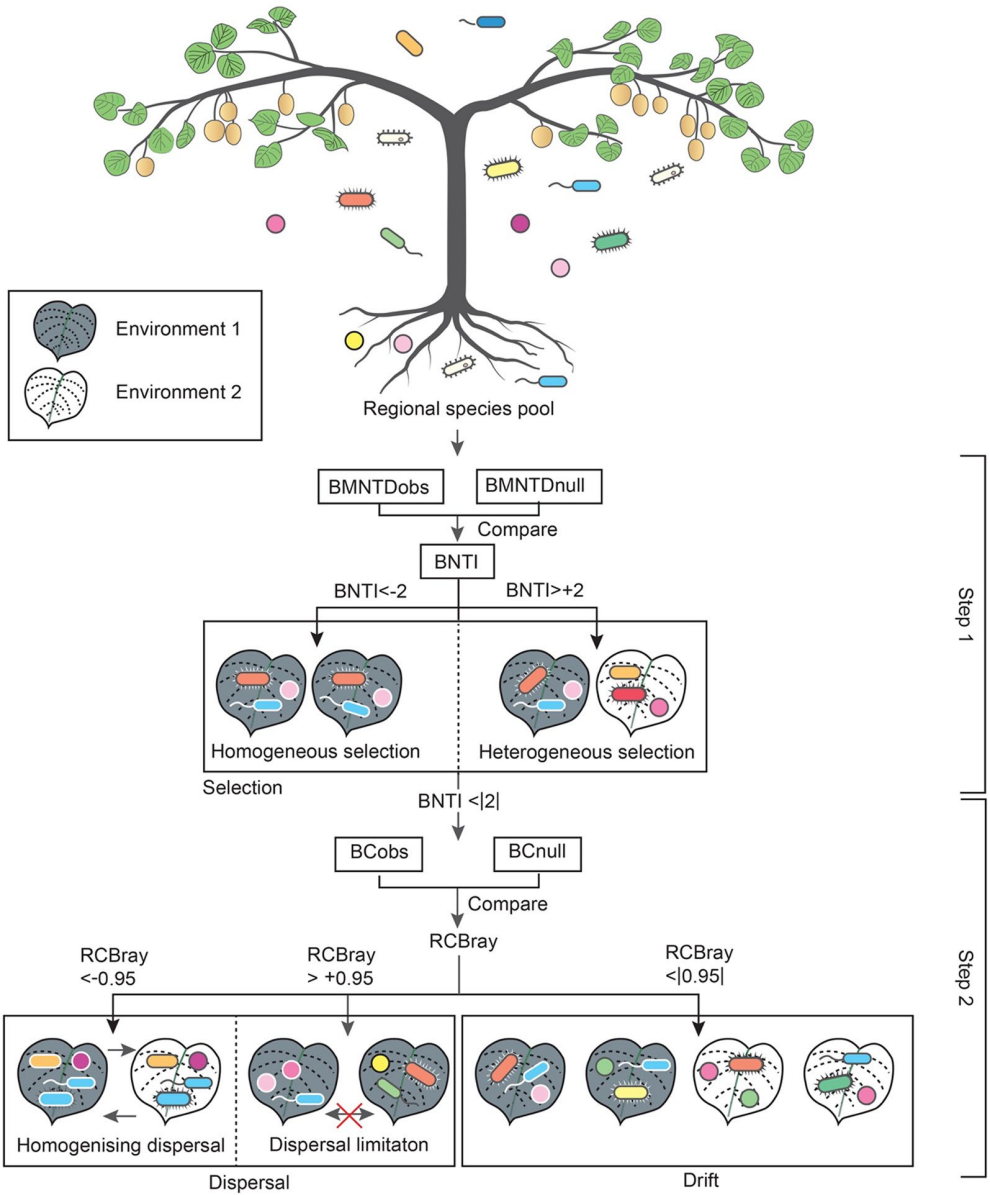
A flow chart displaying the analysis workflow (black arrows) for inferring the influence of ecological processes (box labels) on the kiwifruit vine microbiome, and a summary of the putative assembly processes. The vine signifies the regional microbial species pool or metacommunity. Two environments with different conditions are represented by a grey leaf (environment 1) and a white leaf (environment 2), respectively. For each pair of local communities (run separately for each substrate and time point, n = 60), the observed level of phylogenetic turnover, or beta mean nearest taxon distance, was determined (βMNTDobs). A null distribution of phylogenetic turnover values (βMNTDnull) was computed by randomly shuffling species names and abundances across the tips of the phylogeny. β-nearest taxon index values (βNTI) represent the difference between the βMNTDobs and the mean of the βMNTDnull. A βNTI value of < − 2 or >  + 2 indicates that the community composition was significantly consistent with selection as the assembly process. A βNTI value of < − 2 indicates that homogeneous selection was the principal assembly process. A βNTI value of >  + 2 indicates that heterogeneous selection was the principal assembly process. Further separation of the data was done for samples for which pairwise comparisons were not dominated by either homogeneous or heterogeneous selection (BNTI <|2|). We compared the observed pairwise Bray–Curtis dissimilarity (BCobs) between samples to values obtained from a null distribution of Bray–Curtis values (BCnull). Raup-Crick, based on Bray Curtis dissimilarity (RCBray), uses the deviation between BCobs and BCnull to disentangle variation in community dissimilarity from variation in α-diversity and ranges between − 1 and + 1. An RCBray value of < − 0.95 or >  + 0.95 specifies lower or higher turnover than expected if the sole mechanism shaping the community composition were due to ecological drift. An RCBray value of < − 0.95 indicates that homogenising dispersal was the dominant assembly process. An RCBray value of >  + 0.95 indicates that dispersal limitation was the dominant assembly process. The portion of insignificant RCBray values for each pairwise comparison ( <|0.95|) are those where drift is estimated to dominate community assembly

Figure 1 displays a flowchart summarising our analytical workflow. The first step of the framework involves quantifying the observed β-mean-nearest-taxon-distance (βMNTD), which calculates the phylogenetic turnover between each pair of local communities (n = 60 [5 vines × 12 orchards] per substrate and sampling time). βMNTD was calculated using the *comdist* function from the package ‘picante’ [37]. A null distribution of the phylogenetic turnover was produced through randomisation by shuffling ASV names and abundances across the tips of the phylogenetic tree and recalculating the βMNTD 999 times, simulating expected βMNTD under stochastic processes. The β-nearest taxon index (βNTI) was calculated for each pairwise comparison and represents the difference (in units of one standard deviation of the null distribution) between the observed βMNTD and the mean of the null βMNTD. A βNTI value of < − 2 or >  + 2 indicates that the community composition was significantly consistent with selection as the assembly process.

The second step uses a modified version of the Raup-Crick metric, which incorporates Bray–Curtis distances (RCBray) to further partition the data for samples for which pairwise comparisons were not dominated by selection (βNTI <|2|) into being dominated by homogenising dispersal, dispersal limitation or drift, acting alone. RCBray compares the observed and expected turnover between two local communities, using community composition instead of phylogenetic turnover. A Bray–Curtis dissimilarity is calculated for each pairwise comparison. This observed Bray–Curtis value is compared to a null distribution of Bray–Curtis dissimilarity generated through a randomisation approach where ASVs are probabilistically pulled into each local community until the richness of that community is reached and one individual represents each recruited ASV. The likelihood of drawing a particular ASV is proportional to the total number of sites it occupies. Reads of the recruited ASVs are then drawn into the community until the sample read depth is reached. The probability of being drawn is proportional to that ASV’s relative abundance across all sites. The randomisation process and recalculation of Bray–Curtis dissimilarity was done 999 times to generate the null distribution for each pair of communities. The RCBray metric varies between − 1 and + 1, with a value < − 0.95 or >  + 0.95 specifying lower or higher than expected pairwise similarity in community composition than when drift is acting alone [16, 68]. We separately quantified the percentage contribution of each ecological process across all pairwise comparisons, for each substrate and sampling time.

## Influence of environmental variables

We performed variance partitioning using the *varpart.MEM* function [46] to quantify the relative variance explained by data attributed to the categories of soil chemistry, climate, kiwifruit variety and time on each substrate separately. The soil chemistry (pH, anaerobically mineralisable N: Total N, Total nitrogen (TN, %), C: N, Potassium (me/100 g), Sodium (me/100 g)) and climatic variables (Jan (summer) max temperature (℃), July (winter) min temperature (℃), total precipitation (mm)) were standardised using the *deconstand* function in the R package ‘vegan’ [54]. The kiwifruit variety data of Hayward and Gold were converted into a binary (0/1) variable, representing the two levels of this categorical variable used as model inputs. For the time variable, we represented the months using sine and cosine functions to incorporate the cyclical nature of seasonal variation into the model. To evaluate the relationship between microbial community turnover, climatic variables, and soil chemical properties, we calculated Pearson’s correlations between βNTI and each environmental variable. As we have one composite soil chemical sample per site, we calculated the median βNTI value for each substrate per orchard. The median value represents the median of all pairwise comparisons to every other sample. We found the median value across all times sampled. We plotted the linear regression of those significantly correlated.

## Results

### Variability of bacterial community composition

**Fig. 2:**
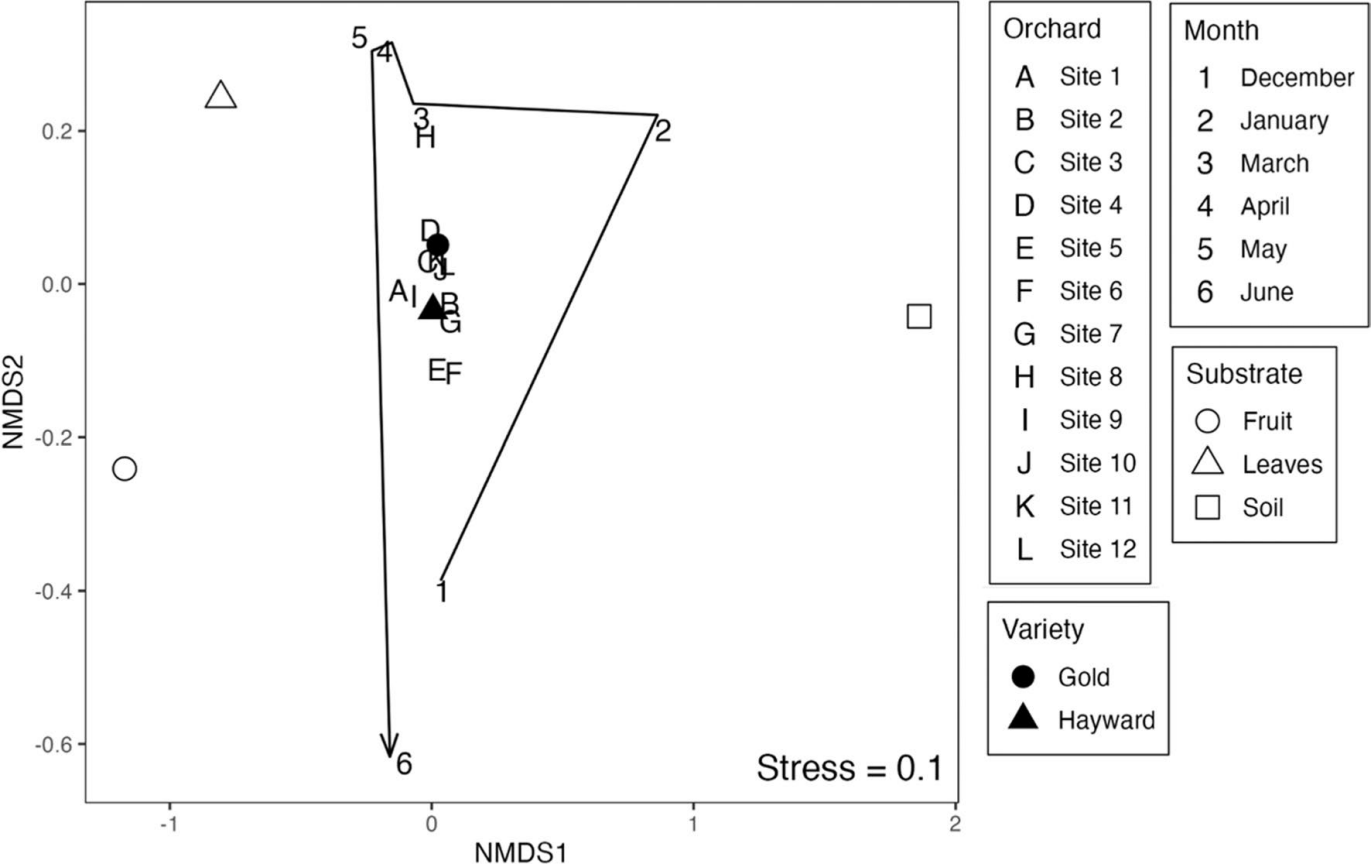
Bray–Curtis dissimilarity-based non-metric multidimensional scaling (NMDS) ordination of bacterial community composition. Differences in bacterial community composition have been averaged to form one representative point for each variable. The trajectory of change in bacterial composition is represented by the arrow connecting the month points

There was no significant relationship between beta diversity (Bray–Curtis) and geographical distance between the orchards (Mantel, r = 0.015, *P* = 0.54). Additionally, bacterial community composition was not significantly different within replicates (PERMANOVA, *P* = 0.518, Additional file 1: Table S3). Bacterial community composition was significantly different among kiwifruit varieties (PERMANOVA *P* < 0.05, Table 1), orchards, substrates (leaves, fruit and soil), and sampling times (PERMANOVA, all *P* < 0.001; Fig. 2, Table 1). Substrate explained the greatest amount of compositional variation (R^2^ = 43%), followed by sampling month (R^2^ = 9%, Table 1). The largest shift in community composition occurred between May and June (Fig. 2). Kiwifruit variety and orchard both explained significant variation in community structure. However, they account for less variation (1% and 4% respectively, Table 1).

**Table 1:** PERMANOVA results of bacterial community composition indicating the partitioning of variation and tests for kiwifruit variety, orchard, sample time and substrate

Source of variation	d.f.	Sum of Sqs	R^2^	F	*P*
Variety	1	0.54	0.01	3.20	< 0.05
Orchard	10	3.22	0.04	1.89	< 0.001
Time	5	6.72	0.09	7.91	< 0.001
Substrate	2	31.23	0.43	91.81	< 0.001
Residuals	185	31.47	0.43

## Greater temporal stability in bacterial community composition in soil than the phyllosphere

**Fig. 3:**
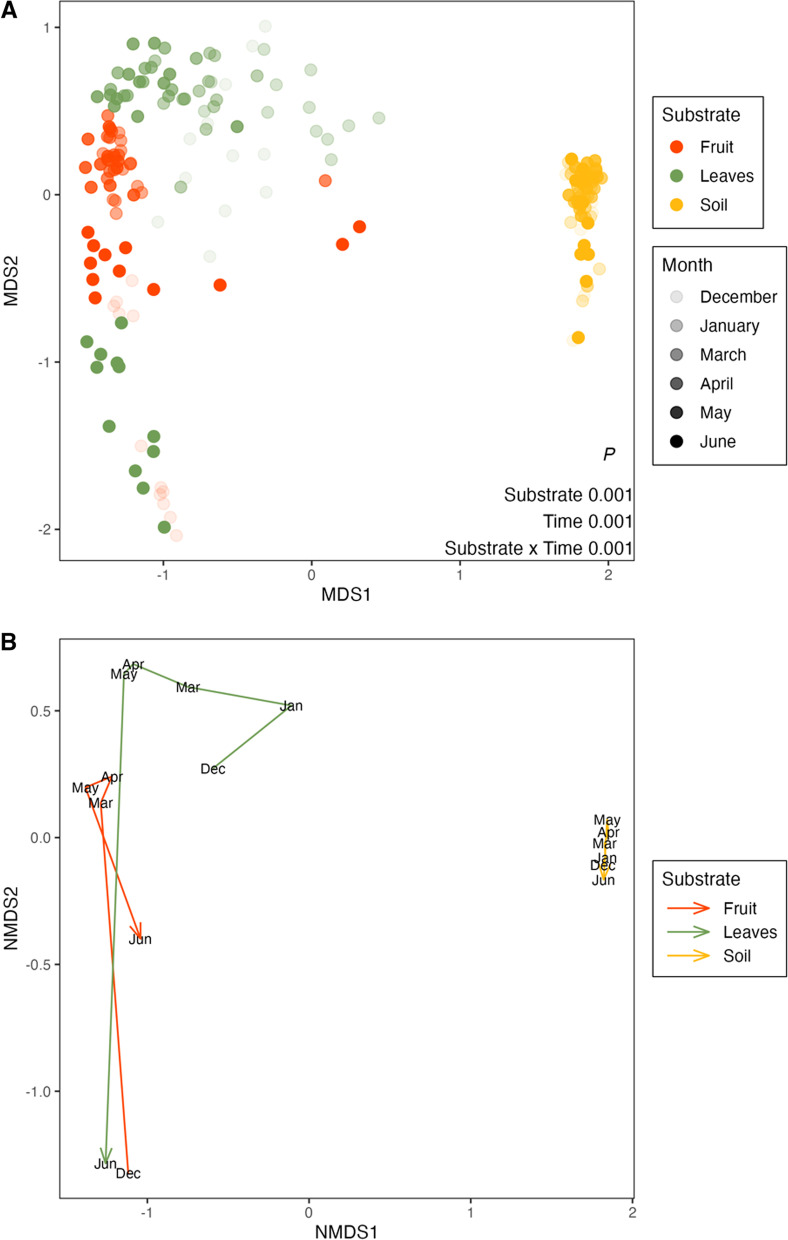
Bray–Curtis dissimilarity-based non-metric multidimensional scaling (NMDS) ordinations of **A** bacterial community composition and **B** the mean trajectory of temporal change in bacterial composition for each substrate. *P*-values from PERMANOVA assessing substrate and temporal effects and their interaction are displayed in the bottom right corner of plot **A**

As most of the variation was attributed to substrate and temporal variation, we plotted two additional NMDS plots to more thoroughly investigate the difference in bacterial community composition among the substrates (Fig. 3a) and their temporal trajectories (Fig. 3b). The greatest difference in community composition was observed between the soil communities and the phyllosphere communities (leaves and fruit) (Fig. 3a). A pairwise PERMANOVA analysis indicated that the community composition among all substrates was significantly different (*P* < 0.001), with 17% of the variation between fruit and leaf communities attributed to substrate, compared to other variation not tested (e.g. time or variety) (Table 2). In comparison, 45% and 42% of the variation between leaf and soil and fruit and soil communities was attributed to substrate (compared to other variation not tested), respectively (Table 2). The Bray–Curtis distances among the sampling times were greater in the leaf and fruit community data compared with the soil community data (Fig. 3). Bacterial composition in the leaf and fruit communities was significantly different between each of the successive sampling times (Pairwise PERMANOVA, *P* < 0.05), except between the March and April fruit samples, which did not significantly differ (Pairwise PERMANOVA, *P* = 0.169) (Additional file 1: Table S4a and b). Bacterial communities within the soil samples were compositionally comparable across the successive sampling times (Pairwise PERMANOVA, *P* > 0.05), except between the samples transitioning from March to April and May to June, which were compositionally distinct from each other (Pairwise PERMANOVA, *P* < 0.05) (Additional file 1: Table S4c). The largest temporal shift in community structure in the leaf data was observed from May to June. The largest temporal shift in community structure in the fruit data was observed from December to March, with the second largest shift observed from May to June (Fig. 3b). Note that we did not have fruit sample data for January; therefore, the December to March transition represents a longer time period.

**Table 2:** Pairwise PERMANOVA testing for the effect of substrate on bacterial community composition

Pairwise comp	d.f	Sum of Sqs	R^2^	F	*P*
Leaves × fruit	1	6.10	0.17	26.17	< 0.001
Leaves × soil	1	21.98	0.45	118.06	< 0.001
Fruit × soil	1	19.06	0.42	95.2	< 0.001

## Taxa identified in the phyllosphere communities were largely not found in the soil communities

**Fig. 4:**
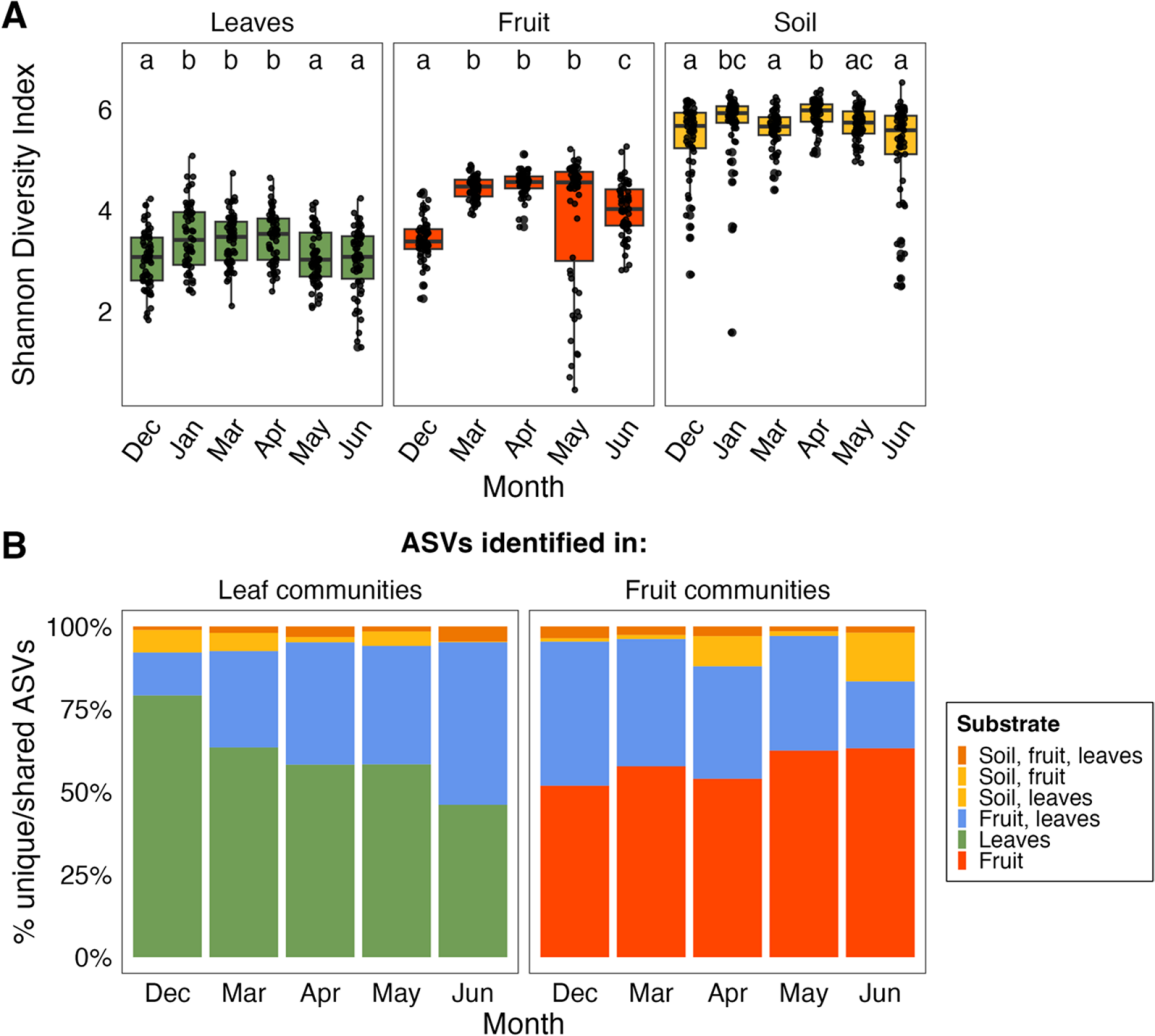
**A** Boxplots displaying the estimated Shannon diversity index values of the bacterial communities of the leaf, fruit and soil substrates. Shannon diversity index values were quantified using the rarefied data. The boxes represent the interquartile range (IQR: 25–75% of the data), the horizontal line indicates the median, while the whiskers extend to 1.5 times the IQR. The points represent individual values for each of the samples. Boxes with different letters within each panel indicate significant differences from each other (Dunn’s *P* < 0.05). **B** Stacked bar plots of the percent of taxa found in the leaf and fruit communities that were only found in data from those substrates or also identified in the data from the other substrates. Bar plots displaying the percent of shared and unique taxa found in the soil communities are displayed in Additional file 1: Fig S5

The most abundant phylum among all the substrates was *Proteobacteria.* Only four abundant phyla, classified as making up over 1% of the total relative abundance, were identified in the phyllosphere communities: *Actinobacteriota Bacteroidota, Firmicutes* and *Proteobacteria.* Contrastingly, there were eight abundant phyla identified in the soil communities: *Acidobacteriota, Actinobacteriota, Bacteroidota, Chloroflexi, Firmicutes, Verrucomicrobio* and *Proteobacteria* (Additional file 1: Fig S4). For each of the substrates, both time and orchard had significant effects (*P* < 0.001) on the alpha diversity of the bacterial communities and a significant interaction (*P* < 0.001) was present. For each substrate, the effect of time was larger than orchard (leaves: *F* = 13.10 and 6.35, fruit: *F* = 39.56 and 5.53, soil: *F* = 12.25 and 6.10 for time and orchard respectively). Overall, bacterial communities in the soil were more diverse than in the phyllosphere communities (Fig. 4a). The bacterial diversity of the leaf communities significantly increased (Shannon index, Dunn’s *P* < 0.05) after January and then decreased in May. There was a significant increase (Dunn’s *P* < 0.05) in bacterial diversity in the fruit samples after December, and then a significant decrease in Shannon diversity in June. The diversity of the bacterial communities in the soil fluctuated through time, with each successive month significantly more or less diverse than the previous month (Dunn’s *P* < 0.05), except May and June where diversity did not significantly differ between the months (Dunn’s *P* > 0.05).

Of the ASVs identified in the phyllosphere samples, the majority were not present in the soil, with > 80% and > 90% of taxa observed in the fruit and leaf samples, respectively, not observed in the soil at each time (Fig. 4). The proportion of unique ASVs observed in the fruit samples remained relatively stable through time, with an average of 57.8% (Fig. 4b). However, the proportion of unique ASVs observed in the leaf samples gradually reduced from 79.2% in December to 46.1% in June. This was largely due to increased ASVs shared with the fruit samples from 13% in December to 49.1% in June (Fig. 4b). Of the ASVs identified in the soil samples, > 90% of taxa observed in the soil samples were not observed in the leaf or fruit communities at each time (Additional file 1: Fig S5).

## Community assembly processes

**Fig. 5:**
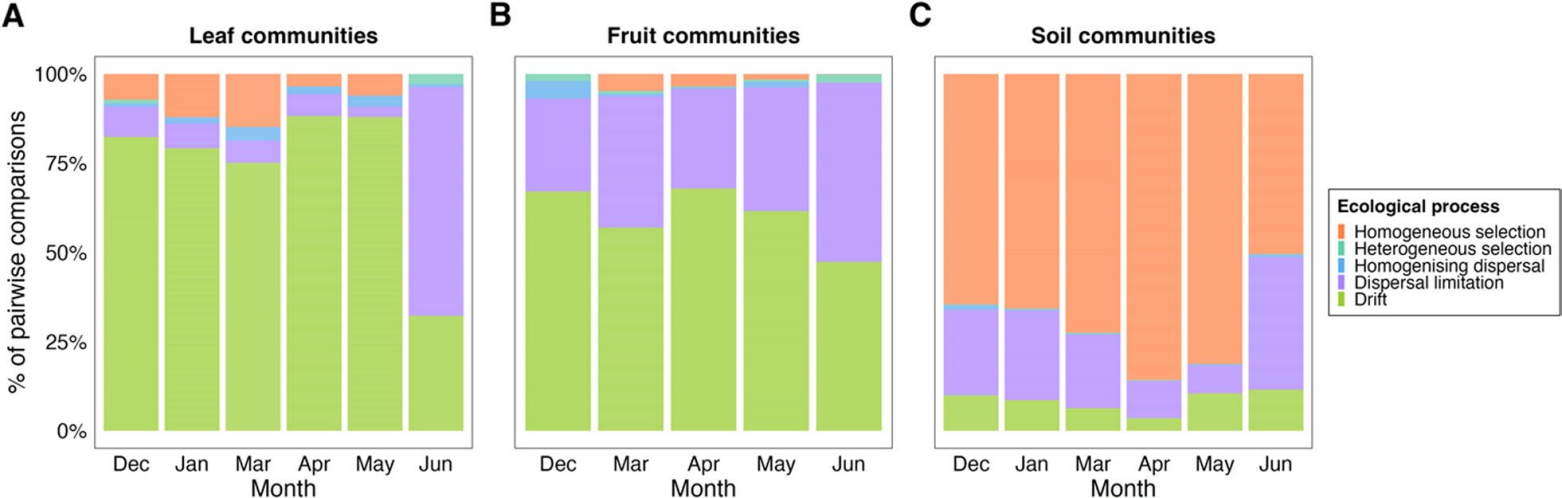
Stacked bar plots of the percent contribution of various ecological processes, determined as the primary assembly process governing the turnover of bacterial communities, for each pairwise comparison between local **A** leaf, **B** fruit and **C** soil communities, and how this varies through time

We identified the dominant community assembly processes in the three substrates and examined how they varied through time. In the phyllosphere substrates, we found stochastic processes were more important than deterministic processes in driving community assembly (largely drift and dispersal limitation, Fig. 5a and b). In the leaf communities, > 70% of all pairwise comparisons for each time, except June, did not have significantly different phylogenetic or compositional turnover than expected if the mechanism shaping turnover were due to ecological drift. In June, the dominant assembly process in the leaf communities shifted from drift (32.2% of all pairwise comparisons) to dispersal limitation (64.1% of all pairwise comparisons; Fig. 5a). The fruit samples had a similar assembly profile to the leaf samples, with drift being the dominant mechanism each time, except June, where it was the dominant process for 47.4% of all pairwise comparisons, while dispersal limitation accounted for 50.2% of all pairwise comparisons (Fig. 5b). In contrast, in the soil communities, deterministic processes were the dominant mechanism driving community assembly (largely homogeneous selection, Fig. 5c). The proportions of estimated ecological processes varied little through time in the soil communities, except for a decrease in homogeneous selection from May to June, from 81.3 to 50.5%, and an increase of dispersal limitation from 7.8 to 37.2% (Fig. 5c).

## Influence of environmental factors

**Fig. 6:**
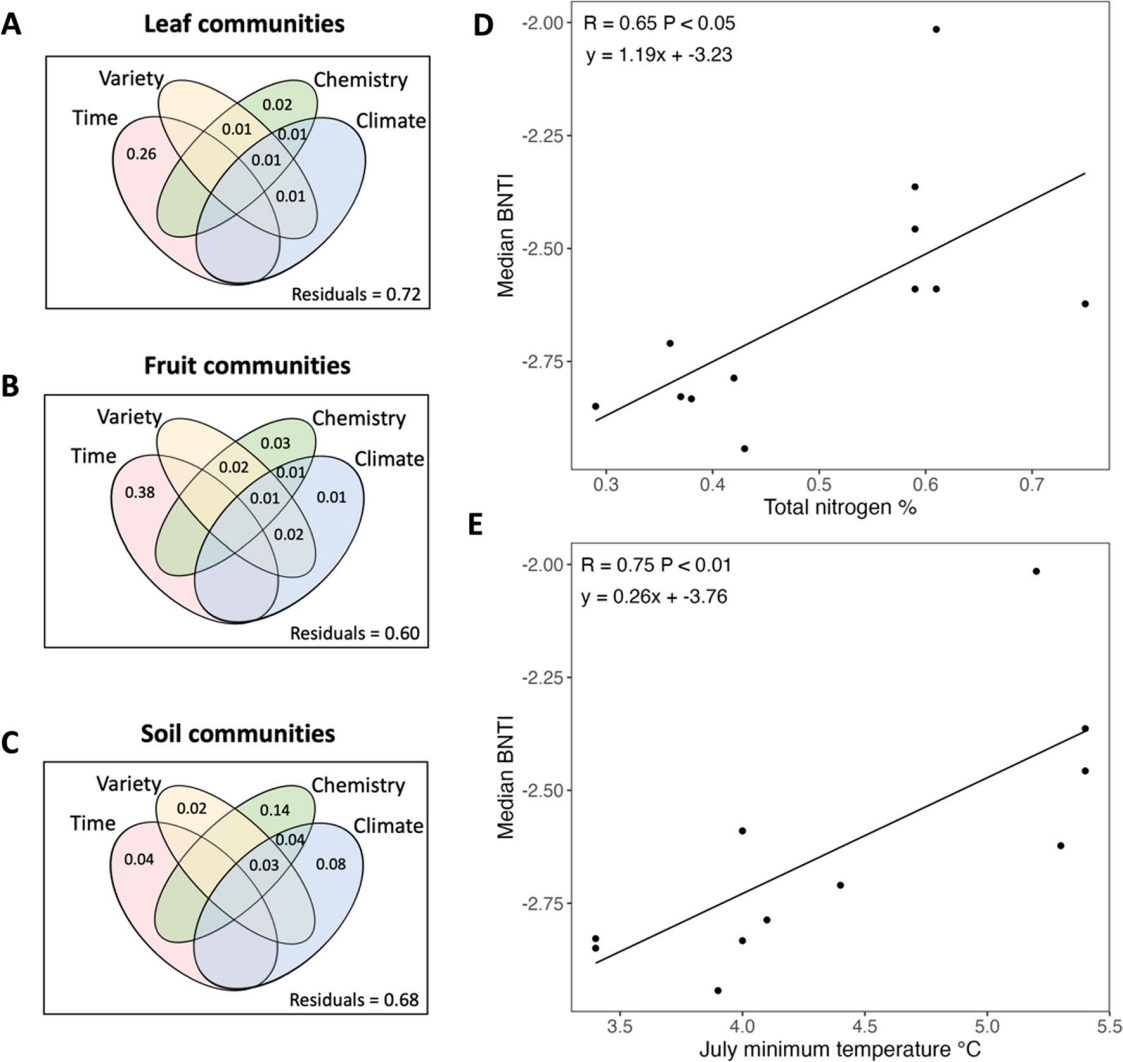
The amount of variation in bacterial community composition of the **A** leaf, **B** fruit and **C** soil communities, based on variance partitioning (VarPart), that could be explained by time (sample dates), kiwifruit variety (Hayward, Gold), soil chemistry (pH, anaerobically mineralisable N: Total N, Total nitrogen (TN, %), C: N, Potassium (me/100 g), Sodium (me/100 g)), climate (Jan (summer) max temperature (℃), July (winter) min temperature (℃), total precipitation (mm)). D & E display the relationship between the median βNTI value of each orchard in the soil communities and **D** total nitrogen and **E** July minimum temperature of each orchard. Pearson’s correlation was used to measure the strength of the relationships

Variance partitioning indicated that the largest variation explained by a single category was time for both the leaf and fruit communities, explaining 26% and 38%, respectively (Fig. 6a and b). For the soil communities, the largest section of variation explained by a single category was 14% by soil chemistry. Soil chemistry explained a further 7% of the variance in community composition when assessed in combination with the other variables (climate; Fig. 6c).

To identify key factors influencing the balance between stochastic and deterministic processes, we assessed the Pearson’s correlation between each environmental variable and the median βNTI value for each substrate per orchard. There were no significant correlations between the measured environmental variables and the median βNTI of the leaf or fruit communities. However, there was a strong relationship between the median βNTI of the soil communities and total nitrogen (slope = 1.19,* R* = 0.65, *P* < 0.05) and a strong relationship between median βNTI and the minimum July (winter) temperature (slope = 0.26, *R* = 0.75, *P* < 0.01; Fig. 6d and e). There were no significant correlations between βNTI of the soil communities and the other measured environmental variables.

## Discussion

Our study provides new insights into the connectivity of soil and phyllosphere bacterial communities in managed systems. Overall, our study indicates very restricted connectivity between the communities residing below and above ground, with distinct community compositions and limited shared taxa. These differences are reflected in the contrasting temporal stability observed and the distinct underlying community assembly processes occurring in the soil and phyllosphere communities. The low connectivity between the belowground and aboveground communities, combined with the high temporal variability and highly stochastic nature of community assembly processes in the phyllosphere environment indicates that predicting the ecological responses to environmental changes or disturbance could be challenging for these communities.

## Soil communities are more stable than phyllosphere communities

We observed significantly different bacterial community compositions among the communities residing on the leaves, fruit and soil substrates, in line with other studies [24, 74, 77, 84]. Additionally, we observed temporal variation in both the diversity and composition of the bacterial communities among all the substrates. However, we showed that the bacterial communities residing in the soil were much more stable through time, relative to the communities located in the phyllosphere. Temporal dynamics of soil microbial communities are highly complex and vary across different land use systems, with both temporal variation [10, 41, 44] and temporal stability [31, 60] observed in agricultural and unmanaged systems. Consistent with our observations, a meta-analysis showed that, when compared to other biomes (e.g. air, flowers, marine), soil bacterial communities had consistently less temporal variation [66]. Our results further support that land use management and soil physiochemical properties are the dominant drivers of community turnover in soil environments [29–31, 43].

In contrast to soil, the bacterial communities on the leaves and fruit were highly variable over time. This is not surprising as we were observing microbial community succession within new environments as the leaves and fruit grow and develop through the season [66]. Although the exposed nature of the phyllosphere will undoubtedly influence community variability, our variance partitioning results suggest that the high temporal variability in the phyllosphere communities is driven to a greater extent by the development of the substrates and community succession than changes in the climate or soil chemical properties.

Furthering our understanding of the seasonal variation in bacterial communities can provide insights into how the communities will respond to disturbances or climate change. The higher temporal stability in the soil communities and the higher diversity may indicate that the soil communities have a greater buffering capacity towards environmental changes than the phyllosphere communities [33]. However, although we have observed relative stability in the soil communities, greater temporal variation could still be present over a longer time-frame than our study (for example, ten years), as soil bacterial communities appear to respond slowly to environmental change [48].

## Limited connectivity between the belowground and aboveground microbial communities

We observed very limited connectivity between the soil microbial communities and the phyllosphere communities, with > 80% and > 90% of taxa observed on the fruit and leaves not present in the soil, respectively. This indicates that alternative sources such as air, surrounding vegetation or animal vectors provide the dominant microbial source populations for phyllosphere communities in kiwifruit orchards. This may also include human vectors as manual operations are carried out in the orchards for pruning and harvesting. Bacterial colonisation of the phyllosphere from the air has been observed [49], however, limited studies focusing on the relative contribution of soil and atmospheric bacteria to phyllosphere communities exist in the field. Sampling the atmospheric microbial communities in addition to the soil and phyllosphere communities can further our understanding of phyllosphere colonisation. Our study suggests the soil communities have reduced influence on phyllosphere colonisation and community turnover. This is in contrast to studies of other agricultural plants where soil has been identified as the major source of phyllosphere microorganisms [24, 27, 85], however it is in agreement with observations from a study examining the strawberry microbiome [55]. These varied results highlight the complexity of microbial communities and suggest host-specific factors and stochastic processes are more important in predicting phyllosphere colonisation than soil properties. In combination with our results of low connectivity between the belowground and aboveground communities, this suggests that management strategies (such as inoculation or pathogen controls) should be considered separately when targeting the soil and phyllosphere components as management practices undertaken in the soil may not necessarily be reflected in the aboveground communities. Alternatively, the differing results could also be an artifact of the different bioinformatic analyses of the sequencing reads. The studies with more connectivity observed used traditional clustering of sequences into operational taxonomic units (OTUs), generally clustered at 97% similarity. However, our sequencing reads and Olimi et al. [55] were resolved to amplicon sequence variants (ASVs) at single-nucleotide resolution [12]. Therefore, our more stringent grouping is potentially why we observed fewer shared taxa (at the ASV level).

## Dominant community assembly processes contrast between the soil and phyllosphere communities

Overall, we found that bacterial communities residing belowground and aboveground were regulated by distinct assembly processes (deterministically dominated in soil communities compared to stochastically dominated in phyllosphere communities). These processes were broadly consistent through time. The strong influence of homogeneous selection on the soil bacterial community assembly was consistent with the larger proportion of community composition explained by soil chemical properties and climatic variables in the variance partitioning analysis. Total soil nitrogen and July minimum temperature were also identified as mediators of deterministic processes in the soil communities. No relationship was determined between the measured environmental variables and the phylogenetic turnover of the phyllosphere communities. The dominance of deterministic processes has been attributed to consistent selective pressure due to consistent environmental conditions [69]. Therefore, the higher consistency in soil environments relative to the phyllosphere likely explains the contrasting assembly processes. Our findings suggest that the responses of bacterial communities to environmental change may be more predictable than those in the phyllosphere, and management strategies targeting soil communities should consider environmental conditions.

Although homogeneous selection was the dominant mechanism, dispersal limitation was also a driver of soil community assembly. Soil pH has been identified as a dominant mediator of the balance between deterministic and stochastic processes, with selection increasing in environments with extreme pH conditions [34, 71]. There was limited variation in the pH levels of the soils in our sites, with relatively neutral pH (average pH = 6.7). The non-extreme chemical conditions of the soils in our sites may explain the influence of stochastic assembly processes as described in Tripathi et al. [71]. However, we expect the influence of dispersal limitation to decrease if we analysed the assembly processes separately for each site, as dispersal limitation has been observed to increase with spatial scale in vineyards [45].

Our findings suggest that stochastic processes such as drift and dispersal underpin the colonisation of the phyllosphere. Dispersal limitation had a higher influence on the structure of communities residing on the fruit, compared to the leaves. In kiwifruit orchards, the fruit hangs below the leaves and is more sheltered from the wind and rain than the exposed leaves. As most bacteria colonising the phyllosphere in our study did not originate from the soil, the more sheltered position of the fruit could explain the higher importance of limited dispersal observed in the fruit communities. Additionally, we saw a large increase in the influence of limited dispersal in the leaf communities in June. Insects play a role in the dispersal of microorganisms, with studies describing the dispersal of *Saccharomyces cerevisiae* by bees [22] and flies [11] in vineyards. Therefore, this increase in the relative importance of dispersal could, in part, be related to reductions in insect vectors as the season transitions to winter [5] and, in turn, reduced bacterial dispersal. However, we do not know the relative influence of insect vectors on phyllosphere colonisation in our orchards, so we cannot draw strong conclusions. Additionally, management practices such as artificial pollination can alter the microbial communities in orchards [39].

The regulation of phyllosphere communities by stochastic processes, combined with the low connectivity between the belowground and the aboveground communities, suggests that predicting community dynamics or pathogen establishment in the phyllosphere could be challenging. Community structure and phylogenetic distance have long been postulated to predict pathogen invasion success due to heightened competitive exclusion from closely related taxa. Therefore, higher diversity can theoretically confer higher resistance to invasion [50, 80]. However, studies vary in the relative importance attributed to phylogenetic relatedness compared with the propagule pressure (the abundance of the invading pathogen) [38, 80]. In our study, the stochastic nature of the community assembly processes observed in the phyllosphere suggests that invasion success by a pathogen will likely depend on propagule pressure rather than strong competition from the resident community. This indicates that manipulating the diversity of the resident community may not be the most successful preventative measure against pathogen invasion, but rather a focus on monitoring or reducing propagule pressure, for example through applying phage-based biocontrol strategies [59].

## Conclusion

As increasing attention is being directed towards potential applications of microbes for higher sustainability and productivity in agriculture, we are seeing a shift from focusing on single beneficial microbes to a more holistic community-level perspective [65]. Therefore, increasing our understanding of the underlying community dynamics and the mechanisms shaping community assembly processes is important, particularly in crop systems. Our findings indicate there is limited connectivity in the belowground and aboveground bacterial communities of kiwifruit plants in terms of common taxa and dominant community assembly processes. This suggests that potential microbiome-targeted management strategies could be more effective when customised to the specific target area, accounting for the contrasting community dynamics.

### Supplementary Information

**Additional file 1**. Supplementary data.

## Data Availability

The amplicon sequence data are available in the NCBI Sequence Read Archive under the accession number PRJNA974875.

## References

[1] Abadi VAJM, Sepehri M, Rahmani HA, Zarei M, Ronaghi A, Taghavi SM, Shamshiripour M (2020). Role of dominant phyllosphere bacteria with plant growth–promoting characteristics on growth and nutrition of maize (*Zea mays* L.). J Soil Sci Plant Nutr.

[2] Abril AB, Torres PA, Bucher EH (2005). The importance of phyllosphere microbial populations in nitrogen cycling in the Chaco semi-arid woodland. J Trop Ecol.

[3] Aguilar P, Sommaruga R (2020). The balance between deterministic and stochastic processes in structuring lake bacterioplankton community over time. Mol Ecol.

[4] Arun K D, Sabarinathan KG, Gomathy M, Kannan R, Balachandar D (2020). Mitigation of drought stress in rice crop with plant growth-promoting abiotic stress-tolerant rice phyllosphere bacteria. J Basic Microbiol.

[5] Bale JS, Hayward SAL (2010). Insect overwintering in a changing climate. J Exp Biol.

[6] Bao L, Cai W, Cao J, Zhang X, Liu J, Chen H (2020). Microbial community overlap between the phyllosphere and rhizosphere of three plants from Yongxing Island, South China sea. Microbiologyopen.

[7] Berg G (2009). Plant–microbe interactions promoting plant growth and health: perspectives for controlled use of microorganisms in agriculture. Appl Microbiol Biotechnol.

[8] Bhattacharjee A, Dubey S, Sharma S (2022). Storage of soil microbiome for application in sustainable agriculture: prospects and challenges. Environ Sci Pollut Res.

[9] Bhattacharyya PN, Jha DK (2012). Plant growth-promoting rhizobacteria (PGPR): emergence in agriculture. World J Microbiol Biotechnol.

[10] Björk RG, Björkman MP, Andersson MX, Klemedtsson L (2008). Temporal variation in soil microbial communities in Alpine tundra. Soil Biol Biochem.

[11] Buser CC, Newcomb RD, Gaskett AC, Goddard MR (2014). Niche construction initiates the evolution of mutualistic interactions. Ecol Lett.

[12] Callahan BJ, McMurdie PJ, Holmes SP (2017). Exact sequence variants should replace operational taxonomic units in marker-gene data analysis. ISME J.

[13] Callahan BJ, Sankaran K, Fukuyama JA, McMurdie PJ, Holmes SP (2016). Bioconductor workflow for microbiome data analysis: from raw reads to community analyses. F1000Research.

[14] Capella-Gutiérrez S, Silla-Martínez JM, Gabaldón T (2009). trimAl: a tool for automated alignment trimming in large-scale phylogenetic analyses. Bioinformatics.

[15] Chaparro JM, Badri DV, Vivanco JM (2014). Rhizosphere microbiome assemblage is affected by plant development. ISME J.

[16] Chase JM, Kraft NJB, Smith KG, Vellend M, Inouye BD (2011). Using null models to disentangle variation in community dissimilarity from variation in α-diversity. Ecosphere.

[17] Chase JM, Myers JA (2011). Disentangling the importance of ecological niches from stochastic processes across scales. Philos Trans R Soc B Biol Sci.

[18] Davis NM, Proctor DM, Holmes SP, Relman DA, Callahan BJ (2018). Simple statistical identification and removal of contaminant sequences in marker-gene and metagenomics data. Microbiome.

[19] Donati I, Cellini A, Sangiorgio D, Vanneste JL, Scortichini M, Balestra GM, Spinelli F (2020). Pseudomonas *syringae* pv. *actinidiae*: ecology, infection dynamics and disease epidemiology. Microb Ecol.

[20] Eichmann R, Richards L, Schäfer P (2021). Hormones as go-betweens in plant microbiome assembly. Plant J.

[21] Van Elsas JD, Chiurazzi M, Mallon CA, Elhottovā D, Krištůfek V, Salles JF (2012). Microbial diversity determines the invasion of soil by a bacterial pathogen. Proc Natl Acad Sci.

[22] Goddard MR, Anfang N, Tang R, Gardner RC, Jun C (2010). A distinct population of *Saccharomyces cerevisiae* in New Zealand: evidence for local dispersal by insects and human-aided global dispersal in oak barrels. Environ Microbiol.

[23] Gong T, Xin X (2021). Phyllosphere microbiota: community dynamics and its interaction with plant hosts. J Integr Plant Biol.

[24] Grady KL, Sorensen JW, Stopnisek N, Guittar J, Shade A (2019). Assembly and seasonality of core phyllosphere microbiota on perennial biofuel crops. Nat Commun.

[25] Graham EB, Knelman JE. Implications of soil microbial community assembly for ecosystem restoration: patterns, process, and potential. Microb Ecol. 2023. 10.1007/s00248-022-02155-w 36735065

[26] Guo F, Zhang T. Biases during DNA extraction of activated sludge samples revealed by high throughput sequencing. Appl Microbiol Biotechnol 2013;97:4607–4616. 10.1007/s00253-012-4244-4 PMC3647099 22760785

[27] Hamonts K, Trivedi P, Garg A, Janitz C, Grinyer J, Holford P (2018). Field study reveals core plant microbiota and relative importance of their drivers. Environ Microbiol.

[28] Hein JW, Wolfe GV, Blee KA (2008). Comparison of rhizosphere bacterial communities in *Arabidopsis thaliana* mutants for systemic acquired resistance. Microb Ecol.

[29] Hermans SM, Buckley HL, Case BS, Curran-cournane F, Taylor M, Lear G (2017). Bacteria as emerging indicators of soil condition. Appl Environ Microbiol.

[30] Hermans SM, Buckley HL, Case BS, Curran-Cournane F, Taylor M, Lear G (2020). Using soil bacterial communities to predict physico-chemical variables and soil quality. Microbiome.

[31] Hermans SM, Buckley HL, Curran-Cournane F, Taylor M, Lear G (2020). Temporal variation in soil bacterial communities can be confounded with spatial variation. FEMS Microbiol Ecol.

[32] Holm S. A simple sequentially rejective multiple test procedure. Scand J Stat. 1979:65–70.

[33] Isbell F, Craven D, Connolly J, Loreau M, Schmid B, Beierkuhnlein C (2015). Biodiversity increases the resistance of ecosystem productivity to climate extremes. Nature.

[34] Jiao S, Lu Y (2020). Soil pH and temperature regulate assembly processes of abundant and rare bacterial communities in agricultural ecosystems. Environ Microbiol.

[35] Jiao S, Yang Y, Xu Y, Zhang J, Lu Y (2020). Balance between community assembly processes mediates species coexistence in agricultural soil microbiomes across eastern China. ISME J.

[36] Katoh K, Standley DM (2013). MAFFT multiple sequence alignment software version 7: improvements in performance and usability. Mol Biol Evol.

[37] Kembel SW, Cowan PD, Helmus MR, Cornwell WK, Morlon H, Ackerly DD (2010). Picante: R tools for integrating phylogenies and ecology. Bioinformatics.

[38] Ketola T, Saarinen K, Lindström L (2017). Propagule pressure increase and phylogenetic diversity decrease community’s susceptibility to invasion. BMC Ecol.

[39] Kim S-H, Do H, Cho G, Kim D-R, Kwak Y-S (2021). Bacterial community structure and the dominant species in imported pollens for artificial pollination. Plant Pathol J.

[40] Kinnunen M, Dechesne A, Albrechtsen H-J, Smets BF (2018). Stochastic processes govern invasion success in microbial communities when the invader is phylogenetically close to resident bacteria. ISME J.

[41] Kivlin SN, Hawkes CV (2016). Temporal and spatial variation of soil bacteria richness, composition, and function in a neotropical rainforest. PLoS ONE.

[42] Klindworth A, Pruesse E, Schweer T, Peplies J, Quast C, Horn M, Glöckner FO (2013). Evaluation of general 16S ribosomal RNA gene PCR primers for classical and next-generation sequencing-based diversity studies. Nucleic Acids Res.

[43] Kostin JE, Cesarz S, Lochner A, Schädler M, Macdonald CA, Eisenhauer N (2021). Land-use drives the temporal stability and magnitude of soil microbial functions and modulates climate effects. Ecol Appl.

[44] Kramer S, Marhan S, Haslwimmer H, Ruess L, Kandeler E (2013). Temporal variation in surface and subsoil abundance and function of the soil microbial community in an arable soil. Soil Biol Biochem.

[45] Larsen S, Albanese D, Stegen J, Franceschi P, Coller E, Zanzotti R, et al. Distinct and temporally stable assembly mechanisms shape bacterial and fungal communities in vineyard soils. Microb Ecol. 2022. 10.1007/s00248-022-02065-x PMC10293400 35835965

[46] Legendre P, Borcard D, Roberts DW (2012). Variation partitioning involving orthogonal spatial eigenfunction submodels. Ecology.

[47] Logares R, Tesson SVM, Canbäck B, Pontarp M, Hedlund K, Rengefors K (2018). Contrasting prevalence of selection and drift in the community structuring of bacteria and microbial eukaryotes. Environ Microbiol.

[48] Louisson Z, Hermans SM, Buckley HL, Case BS, Taylor M, Curran-Cournane F, Lear G (2023). Land use modification causes slow, but predictable, change in soil microbial community composition and functional potential. Environ Microbiome.

[49] Maignien L, DeForce EA, Chafee ME, Eren AM, Simmons SL (2014). Ecological succession and stochastic variation in the assembly of *Arabidopsis thaliana* phyllosphere communities. MBio.

[50] Mason NWH, Orwin K, Lambie S, Woodward SL, McCready T, Mudge P (2016). Leaf economics spectrum–productivity relationships in intensively grazed pastures depend on dominant species identity. Ecol Evol.

[51] McMurdie PJ, Holmes S (2013). Phyloseq: an R package for reproducible interactive analysis and graphics of microbiome census data. PLoS ONE.

[52] Mus F, Crook MB, Garcia K, Garcia Costas A, Geddes BA, Kouri ED (2016). Symbiotic nitrogen fixation and the challenges to its extension to nonlegumes. Appl Environ Microbiol.

[53] Nemergut DR, Schmidt SK, Fukami T, O’Neill SP, Bilinski TM, Stanish LF (2013). Patterns and processes of microbial community assembly. Microbiol Mol Biol Rev.

[54] Oksanen J, Blanchet FG, Friendly M, Kindt R, Legendre P, Mcglinn D, et al. Vegan: community ecology package. R Packag version 2020: 25–7.

[55] Olimi E, Kusstatscher P, Wicaksono WA, Abdelfattah A, Cernava T, Berg G (2022). Insights into the microbiome assembly during different growth stages and storage of strawberry plants. Environ Microbiome.

[56] Ottesen AR, Gorham S, Reed E, Newell MJ, Ramachandran P, Canida T (2016). Using a control to better understand phyllosphere microbiota. PLoS ONE.

[57] Paradis E, Schliep K (2019). ape 5.0: an environment for modern phylogenetics and evolutionary analyses in R. Bioinformatics.

[58] Paulson JN, Stine OC, Bravo HC, Pop M (2013). Differential abundance analysis for microbial marker-gene surveys. Nat Methods.

[59] Pereira C, Costa P, Pinheiro L, Balcão VM, Almeida A (2021). Kiwifruit bacterial canker: an integrative view focused on biocontrol strategies. Planta.

[60] Pereira e Silva MC, Dias ACF, van Elsas JD, Salles JF (2012). Spatial and temporal variation of archaeal, bacterial and fungal communities in agricultural soils. PLoS ONE.

[61] Philippot L, Raaijmakers JM, Lemanceau P, van der Putten WH (2013). Going back to the roots: the microbial ecology of the rhizosphere. Nat Rev Microbiol.

[62] Price MN, Dehal PS, Arkin AP (2010). FastTree 2–approximately maximum-likelihood trees for large alignments. PLoS ONE.

[63] Quast C, Pruesse E, Yilmaz P, Gerken J, Schweer T, Yarza P (2013). The SILVA ribosomal RNA gene database project: improved data processing and web-based tools. Nucleic Acids Res.

[64] R Core Team (2023). R: a language and environment for statistical computing.

[65] Ray P, Lakshmanan V, Labbé JL, Craven KD (2020). Microbe to microbiome: a paradigm shift in the application of microorganisms for sustainable agriculture. Front Microbiol.

[66] Shade A, Gregory Caporaso J, Handelsman J, Knight R, Fierer N (2013). A meta-analysis of changes in bacterial and archaeal communities with time. ISME J.

[67] de Souza RSC, Okura VK, Armanhi JSL, Jorrín B, Lozano N, Da Silva MJ (2016). Unlocking the bacterial and fungal communities assemblages of sugarcane microbiome. Sci Rep.

[68] Stegen JC, Lin X, Fredrickson JK, Chen X, Kennedy DW, Murray CJ (2013). Quantifying community assembly processes and identifying features that impose them. ISME J.

[69] Stegen JC, Lin X, Fredrickson JK, Konopka AE (2015). Estimating and mapping ecological processes influencing microbial community assembly. Front Microbiol.

[70] Stegen JC, Lin X, Konopka AE, Fredrickson JK (2012). Stochastic and deterministic assembly processes in subsurface microbial communities. ISME J.

[71] Tripathi BM, Stegen JC, Kim M, Dong K, Adams JM, Lee YK (2018). Soil pH mediates the balance between stochastic and deterministic assembly of bacteria. ISME J.

[72] Trivedi P, Leach JE, Tringe SG, Sa T, Singh BK (2020). Plant–microbiome interactions: from community assembly to plant health. Nat Rev Microbiol.

[73] Turner TR, James EK, Poole PS (2013). The plant microbiome. Genome Biol.

[74] Ueda Y, Frindte K, Knief C, Ashrafuzzaman MD, Frei M (2016). Effects of elevated tropospheric ozone concentration on the bacterial community in the phyllosphere and rhizoplane of rice. PLoS ONE.

[75] Vanneste JL (2017). The scientific, economic, and social impacts of the New Zealand outbreak of bacterial canker of kiwifruit (Pseudomonas syringae pv. actinidiae). Annu Rev Phytopathol.

[76] Vellend M (2010). Conceptual synthesis in community ecology. Q Rev Biol.

[77] Wagner MR, Roberts JH, Balint-Kurti P, Holland JB (2020). Heterosis of leaf and rhizosphere microbiomes in field-grown maize. New Phytol.

[78] Wang K, Yan H, Peng X, Hu H, Zhang H, Hou D (2020). Community assembly of bacteria and archaea in coastal waters governed by contrasting mechanisms: a seasonal perspective. Mol Ecol.

[79] Wang Q, Garrity GM, Tiedje JM, Cole JR (2007). Naïve Bayesian classifier for rapid assignment of rRNA sequences into the new bacterial taxonomy. Appl Environ Microbiol.

[80] Wei Z, Yang T, Friman V-P, Xu Y, Shen Q, Jousset A (2015). Trophic network architecture of root-associated bacterial communities determines pathogen invasion and plant health. Nat Commun.

[81] Weiss S, Xu ZZ, Peddada S, Amir A, Bittinger K, Gonzalez A (2017). Normalization and microbial differential abundance strategies depend upon data characteristics. Microbiome.

[82] Whipps JM (2001). Microbial interactions and biocontrol in the rhizosphere. J Exp Bot.

[83] Wratt DS, Tait A, Griffiths G, Espie P, Jessen M, Keys J (2006). Climate for crops: integrating climate data with information about soils and crop requirements to reduce risks in agricultural decision-making. Meteorol Appl.

[84] Yang H, Zheng Y, Yang Z, Wang Q-C, Lü P-P, Hu H-W (2023). Bacterial communities in the phyllosphere are distinct from those in root and soil, and sensitive to plant species changes in subtropical tree plantations. FEMS Microbiol Ecol.

[85] Zarraonaindia I, Owens SM, Weisenhorn P, West K, Hampton-Marcell J, Lax S (2015). The soil microbiome influences grapevine-associated microbiota. MBio.

[86] Zhalnina K, Louie KB, Hao Z, Mansoori N, Da Rocha UN, Shi S (2018). Dynamic root exudate chemistry and microbial substrate preferences drive patterns in rhizosphere microbial community assembly. Nat Microbiol.

[87] Zhao Z, Ma Y, Feng T, Kong X, Wang Z, Zheng W, Zhai B (2022). Assembly processes of abundant and rare microbial communities in orchard soil under a cover crop at different periods. Geoderma.

[88] Zhou J, Ning D (2017). Stochastic community assembly: Does it matter in microbial ecology?. Microbiol Mol Biol Rev.

