# Supplementary material for: Soil bacterial community composition is more stable in kiwifruit orchards relative to phyllosphere communities over time
Source: Environ Microbiome. 2023 Aug 24;18:71. doi: 10.1186/s40793-023-00526-5 (PMC10463660; doi:10.1186/s40793-023-00526-5)
Supplement: Supplementary file 1 — Additional file 1. Supplementary data. [file 40793_2023_526_MOESM1_ESM.docx]

**
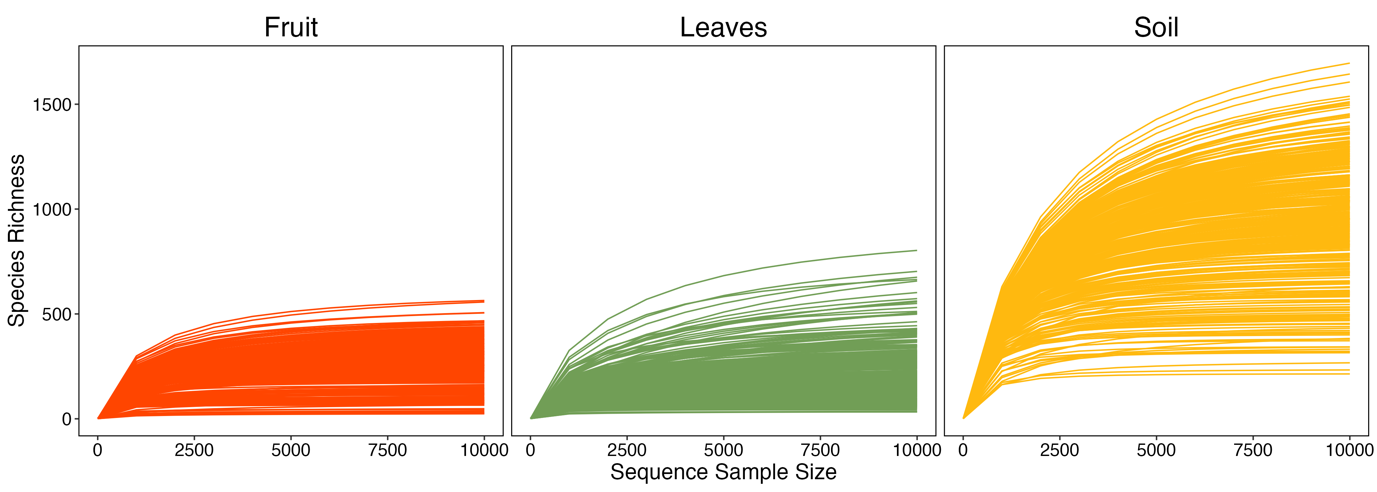
**

**Figure S1:** Rarefaction curves of the ASV data, displaying the relative taxon richness of bacterial communities from the analysis of up to 10,000 16S rRNA gene sequences, grouped by the substrate.


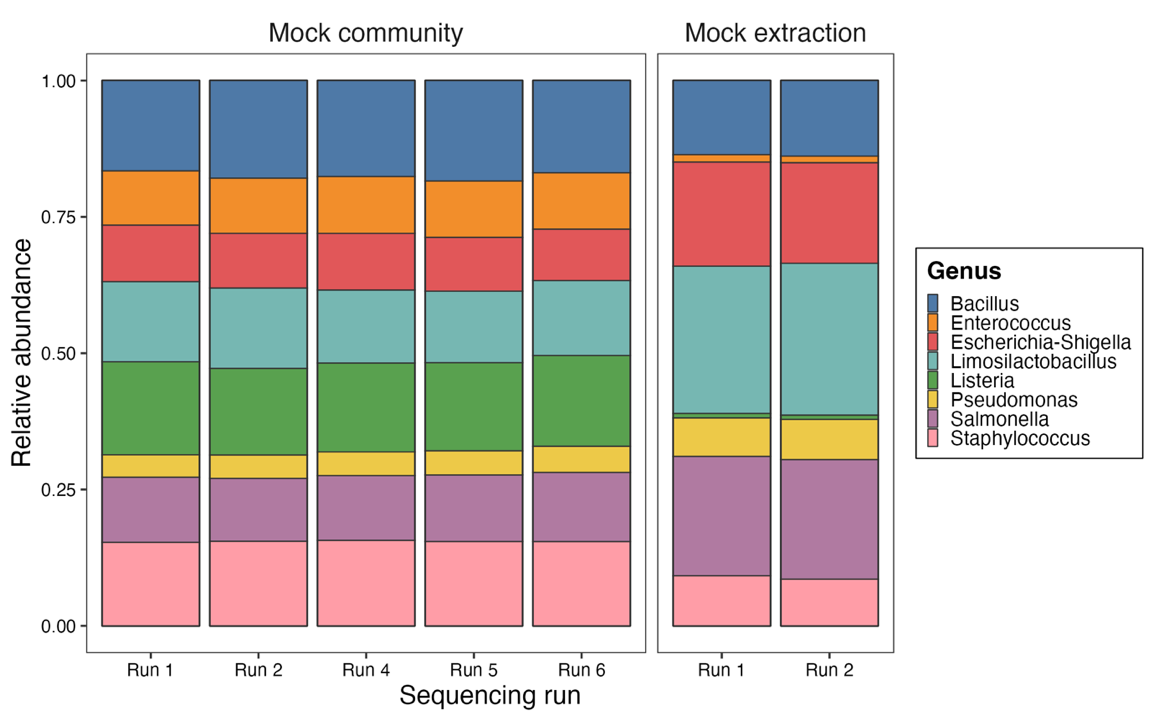


**Figure S2:** Stacked bar plots of the relative abundance of the genera identified in our mock samples. Sequencing run three was omitted as no reads were retained after merging the paired-end reads

**
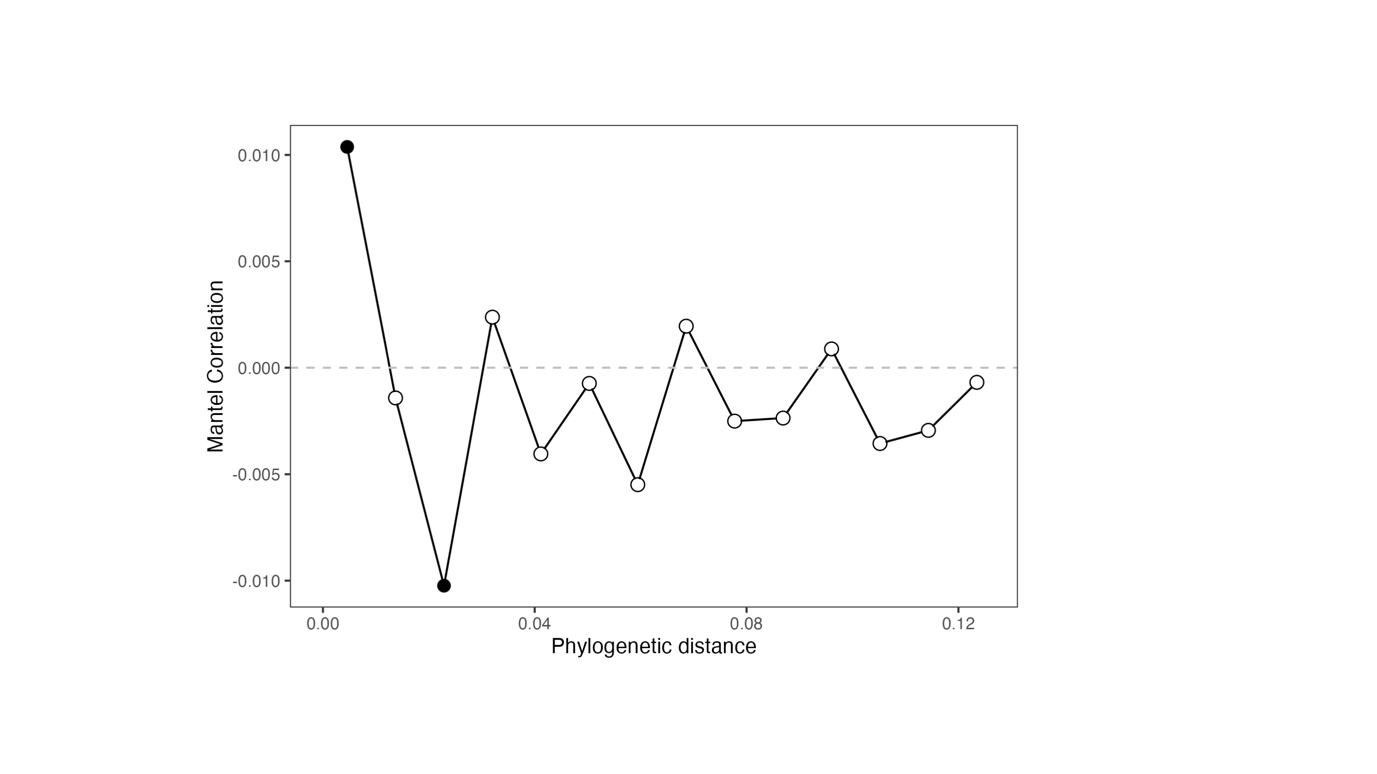
**

**Figure S3:** Phylogenetic signal was tested by quantifying the relative abundance-weighted mean for each ASV based on all non-correlating soil chemical and climatic data and represented as a Euclidean distance matrix to signify niche preferences of each ASV. Phylogenetic distance was calculated based on the branch length of the phylogenetic tree. A Mantel correlogram was used to test this relationship with a significant phylogenetic signal found across short phylogenetic distance, indicating niche distance increases with phylogenetic distance. Solid squares indicate significant correlations, while open squares indicate nonsignificant correlations between ASV niche preferences and phylogenetic distances.


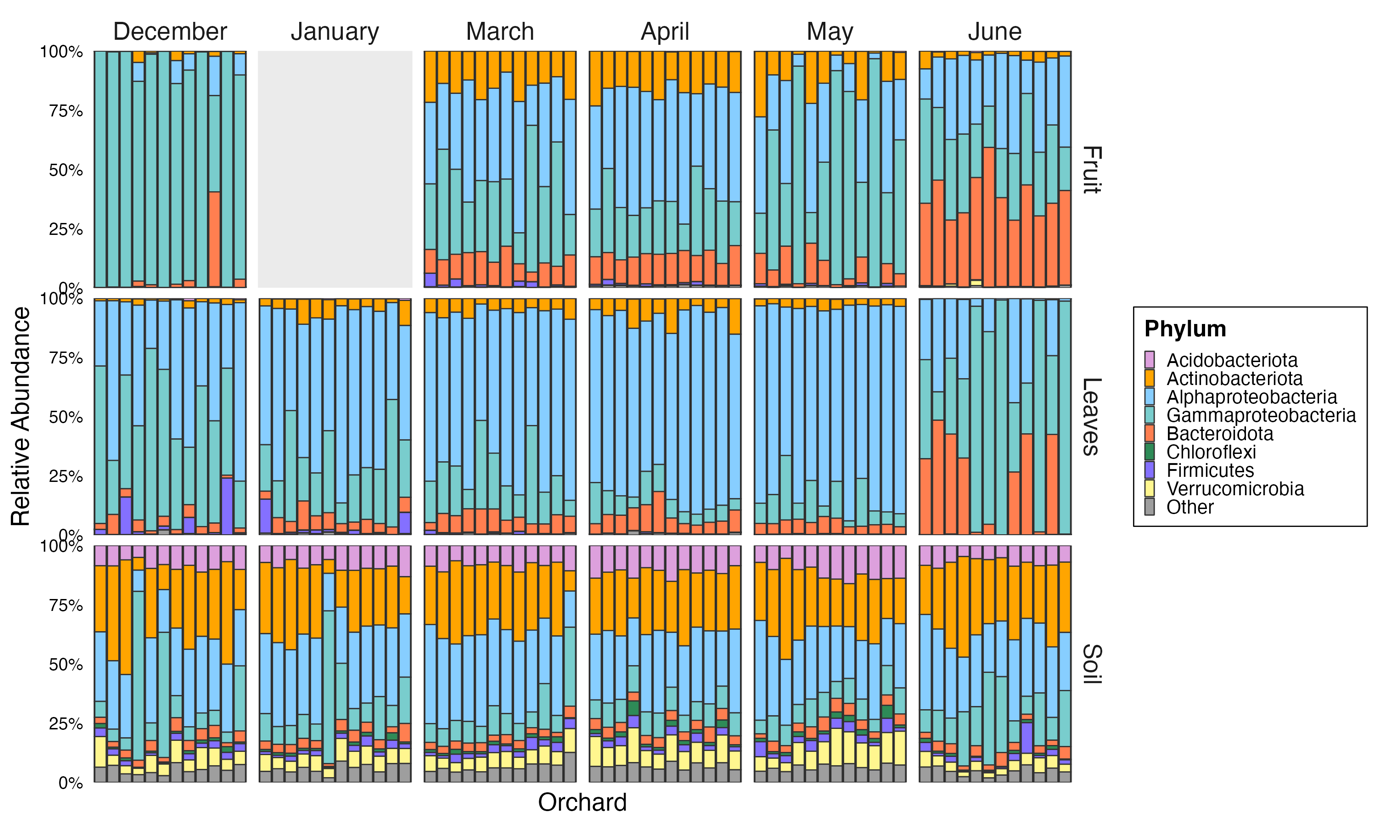


**Figure S4:** Stacked bar plots of the relative abundance of the abundant phyla (representing >1% of the total relative abundance), with bacteria grouped by phyla, except Proteobacteria, which are split by their respective classes.

**
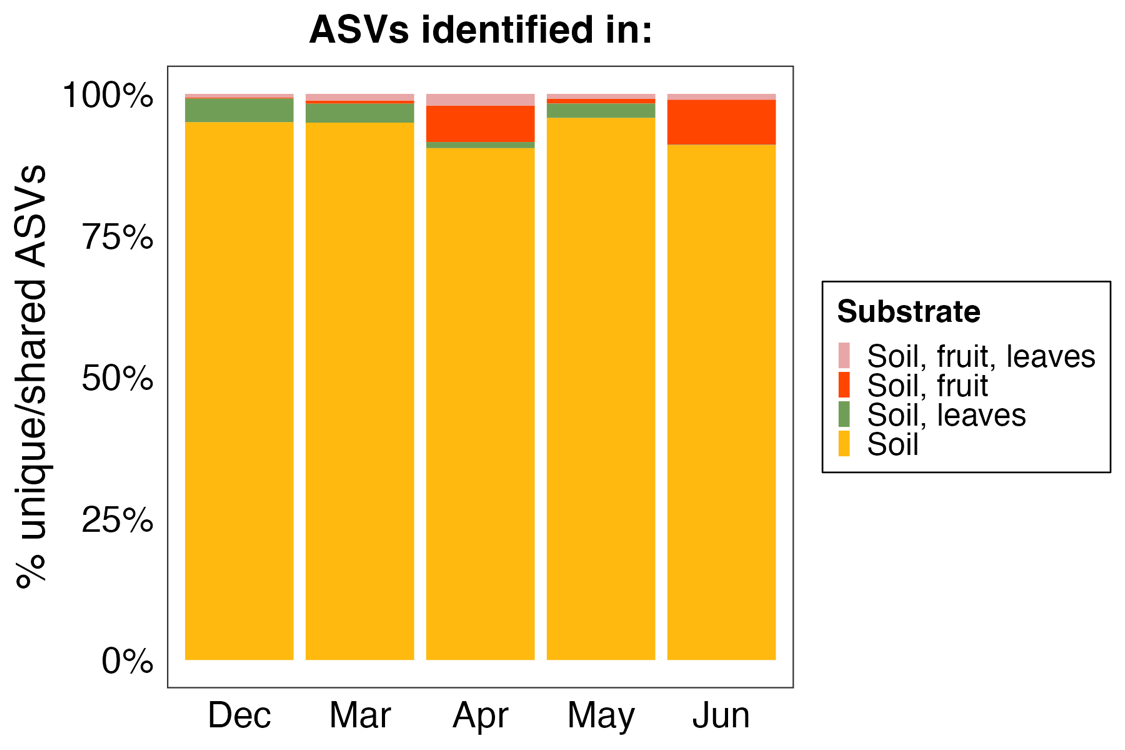
**

**Figure S5** Stacked bar plots of the percent of taxa found in the soil communities that were only found in data from those substrates or also identified in the data from the other substrates.

**Supplementary tables**

**Table S1:** Summary of methods used to conduct analyses for each explanatory analysed by Hill Laboratories, Hamilton, New Zealand

| **Test** | **Method description** |
| --- | --- |
| pH | 1:2 (v/v) soil:water slurry followed by potentiometric determination of pH |
| Olsen Phosphorus | Olsen extraction followed by Molybdenum Blue colourimetry |
| Potentially Available Nitrogen | Anaerobic incubation followed by extraction using 2M KCl 1-12 followed by Berthelot colourimetry. |
| Anaerobically Mineralisable N | As for Potentially Available Nitrogen, but reported as µg/g. |
| Anaerobically Mineralisable N/Total N Ratio | Any Mineral N present is included in the AN/AMN result reported |
| Organic Matter | Organic Matter is 1.72 x Total Carbon. |
| Total Carbon | Dumas combustion |
| Total Nitrogen | Dumas combustion |
| Carbon to Nitrogen | Carbon to nitrogen ratio |
| Potassium | 1M Neutral ammonium acetate extraction followed by ICP-OES |
| Calcium | 1M Neutral ammonium acetate extraction followed by ICP-OES |
| Magnesium | 1M Neutral ammonium acetate extraction followed by ICP-OES |
| Sodium | 1M Neutral ammonium acetate extraction followed by ICP-OES |
| Cation Exchange 1-12 Capacity | Summation of extractable cations (K, Ca, Mg, Na) and 1-12 extractable acidity |
| Total Base Saturation | Calculated from Extractable Cations and Cation Exchange Capacity. |

**Table S2:** Explanatory variables used to explain sources of variation in bacterial community composition. Variables in the same column, with the same letter (superscript) correlated with each other (Pearson’s correlation >0.65 or <-0.65).

|  | **Climate** | **Soil** |
| --- | --- | --- |
| **Variables included** | Jan (Summer) max temperature (℃)^a^ , July (Winter) min temperature (℃)^b^ , Total precipitation (mm)^c^ | pH^a^, Anaerobically Mineralisable N:Total N, Total nitrogen (TN, %)^b^, C:N, Potassium (me/100g), Sodium (me/100g) %) |
|  |  |  |
| **Variables omitted due to correlations** | Mean annual temperature (℃)^a^ , Annual rain days^b^, Winter precipitation (mm)^b^ Summer precipitation (mm)^c^, | Olsen_P (mg/L)^a^, Base saturation (%)^a^,  Potentially Available N (kg/ha)^b^, Anaerobically Mineralisable N (µg/g)^b^, Organic matter (%)^b^, Total Carbon (%), Calcium (me/100g) ^b^, Cation Exchange Capacity ^b^, Magnesium (me/100g)^b^ |
|  |  |  |
| **Data source** | Extracted from NIWA interpolations of climate station data (Wratt et al., 2006) | Composite soil samples (Hill Laboratories, NZ) |

**Table S3:** PERMANOVA results of bacterial community composition (with separate replicates), indicating the partitioning of variation and tests for replicates, orchard, sample time and substrate.

| **Source of variation** | **d .f.** | **Sum of Sqs** | **R^2^** | **F** | ***P*** |
| --- | --- | --- | --- | --- | --- |
| Replicate | 4 | 0.82 | 0.002 | 0.944 | 0.518 |
| Orchard | 11 | 14.21 | 0.035 | 5.993 | 0.001 |
| Time | 5 | 27.35 | 0.067 | 25.117 | 0.001 |
| Substrate | 2 | 133.12 | 0.328 | 305.635 | 0.001 |
| Residual | 1057 | 230.20 | 0.567 |  |  |

**Table S4:** Pairwise PERMANOVA testing for the effect of time on the bacterial composition of **A** leaf, **B** fruit and **C** soil communities.

**A** Leaf community data.

| **Pairwise comp** | **d.f.** | **Sum of Sqs** | **R^2^** | **F** | ***P*** |
| --- | --- | --- | --- | --- | --- |
| Leaf: Dec x Jan | 1 | 0.65 | 0.17 | 4.36 | **0.001** |
| Leaf: Jan x Mar | 1 | 0.30 | 0.10 | 2.48 | **0.003** |
| Leaf: Mar x Apr | 1 | 0.22 | 0.09 | 2.25 | **0.019** |
| Leaf: Apr x May | 1 | 0.46 | 0.19 | 5.01 | **0.001** |
| Leaf: May x Jun | 1 | 2.28 | 0.36 | 12.26 | **0.001** |

**B** Fruit community data

| **Pairwise comp** | **d.f.** | **Sum of Sqs** | **R^2^** | **F** | ***P*** |
| --- | --- | --- | --- | --- | --- |
| Fruit: Dec x Mar | 1 | 2.29 | 0.44 | 17.01 | **0.001** |
| Fruit: Mar x Apr | 1 | 0.14 | 0.06 | 1.307 | 0.189 |
| Fruit: Apr x May | 1 | 0.53 | 0.12 | 3.029 | **0.005** |
| Fruit: May x Jun | 1 | 1.41 | 0.23 | 6.60 | **0.001** |

**C** Soil community data

| **Pairwise comp** | **d.f.** | **Sum of Sqs** | **R^2^** | **F** | ***P*** |
| --- | --- | --- | --- | --- | --- |
| Soil: Dec x Jan | 1 | 0.15 | 0.04 | 0.88 | 0.516 |
| Soil: Jan x Mar | 1 | 0.12 | 0.04 | 0.88 | 0.578 |
| Soil: Mar x Apr | 1 | 0.19 | 0.07 | 1.77 | **0.036** |
| Soil: Apr x May | 1 | 0.10 | 0.04 | 0.94 | 0.454 |
| Soil: May x Jun | 1 | 0.55 | 0.13 | 3.40 | **0.005** |
